# Pitfalls in genetic testing: the story of missed *SCN1A* mutations

**DOI:** 10.1002/mgg3.217

**Published:** 2016-04-14

**Authors:** Tania Djémié, Sarah Weckhuysen, Sarah von Spiczak, Gemma L. Carvill, Johanna Jaehn, Anna‐Kaisa Anttonen, Eva Brilstra, Hande S. Caglayan, Carolien G. de Kovel, Christel Depienne, Eija Gaily, Elena Gennaro, Beatriz G. Giraldez, Padhraig Gormley, Rosa Guerrero‐López, Renzo Guerrini, Eija Hämäläinen, Corinna Hartmann, Laura Hernandez‐Hernandez, Helle Hjalgrim, Bobby P. C. Koeleman, Eric Leguern, Anna‐Elina Lehesjoki, Johannes R. Lemke, Costin Leu, Carla Marini, Jacinta M. McMahon, Davide Mei, Rikke S. Møller, Hiltrud Muhle, Candace T. Myers, Caroline Nava, Jose M. Serratosa, Sanjay M. Sisodiya, Ulrich Stephani, Pasquale Striano, Marjan J. A. van Kempen, Nienke E. Verbeek, Sunay Usluer, Federico Zara, Aarno Palotie, Heather C. Mefford, Ingrid E. Scheffer, Peter De Jonghe, Ingo Helbig, Arvid Suls, EuroEPINOMICS‐RES Dravet working group

**Affiliations:** ^1^ Neurogenetics groupDepartment of Molecular GeneticsVIBAntwerpBelgium; ^2^ Laboratory of NeurogeneticsInstitute Born‐BungeUniversity of AntwerpAntwerpBelgium; ^3^ Sorbonne universitésUPMC université Paris 0691‐105boulevard de l'HôpitalParis75013France; ^4^ ICM, CNRS UMR 7225, Inserm U 112747/83, boulevard de l'HôpitalParis75013France; ^5^ Centre de reference épilepsies raresEpilepsy unit, AP‐HP Groupe hospitalier Pitié‐SalpêtrièreParis75013France; ^6^ Department of NeuropediatricsUniversity Medical Center Schleswig‐HolsteinKielGermany; ^7^ Division of Genetic MedicineDepartment of PediatricsUniversity of WashingtonSeattleWashington98195USA; ^8^ Folkhälsan Institute of GeneticsHelsinkiFinland; ^9^ Medical and Clinical GeneticsUniversity of HelsinkiHelsinkiFinland; ^10^ Helsinki University HospitalHelsinkiFinland; ^11^ Department of Medical GeneticsUniversity Medical Center UtrechtUtrechtThe Netherlands; ^12^ Department of Molecular Biology and GeneticsBogaziçi UniversityIstanbulTurkey; ^13^ Département de génétiqueAP‐HP, hôpital Pitié‐Salpêtrière47/83boulevard de l'HôpitalParis75013France; ^14^ Department of Pediatric NeurologyHelsinki University HospitalHelsinkiFinland; ^15^ Laboratory of GeneticsE.O. Ospedali GallieraGenovaItaly; ^16^ Neurology Laboratory and Epilepsy UnitDepartment of NeurologyInstituto de Investigatión Sanitaria‐Fundación Jiménez Díaz, Universidad Autónoma de MadridMadridSpain; ^17^ IIS‐Fundación Jiménez Díaz and Centro de Investigación Biomédica en Red de Enfermedades Raras (CIBERER)MadridSpain; ^18^ Psychiatric and Neurodevelopmental Genetics UnitMassachusetts General Hospital and Harvard Medical SchoolBostonMassachusetts02114USA; ^19^ Program in Medical and Population GeneticsBroad Institute of MIT and HarvardCambridgeMassachusetts02142USA; ^20^ Stanley Center for Psychiatric ResearchBroad Institute of MIT and HarvardCambridgeMassachusetts02142USA; ^21^ Wellcome Trust Sanger InstituteHinxtonUnited Kingdom; ^22^ Pediatric Neurology and Neurogenetics Unit and LaboratoriesA. Meyer Children's Hospital‐University of FlorenceFlorenceItaly; ^23^ Institute for Molecular Medicine Finland FIMMUniversity of HelsinkiHelsinkiFinland; ^24^ Department of Clinical and Experimental EpilepsyNIHR University College London Hospitals Biomedical Research CentreUCL Institute of NeurologyLondonUnited Kingdom; ^25^ The Epilepsy SocietyChalfont‐St‐PeterBucksUnited Kingdom; ^26^ Department of NeurologyDanish Epilepsy CentreDianalundDenmark; ^27^ Institute for Regional Health researchUniversity of Southern DenmarkOdenseDenmark; ^28^ Research Programs UnitMolecular NeurologyUniversity of HelsinkiHelsinkiFinland; ^29^ Institute of Human GeneticsUniversity of LeipzigLeipzigGermany; ^30^ Epilepsy Research CentreDepartment of MedicineUniversity of MelbourneAustin HealthMelbourneAustralia; ^31^ Pediatric Neurology and Muscular Diseases UnitDepartment of NeurosciencesRehabilitationOphthalmologyGeneticsand Maternal and Child HealthUniversity of Genoa ‘G. Gaslini’ InstituteGenovaItaly; ^32^ Laboratory of NeurogeneticsDepartment of NeurosciencesGiannina Gaslini InstituteGenovaItaly; ^33^ Department of PaediatricsUniversity of Melbourne and Royal Children's HospitalParkvilleVictoria3052Australia; ^34^ Florey Institute of Neuroscience and Mental HealthMelbourneVictoria3084Australia; ^35^ Division of NeurologyAntwerp University HospitalAntwerpBelgium; ^36^ Division of NeurologyChildren's Hospital of PhiladelphiaPhiladelphiaPennsylvaniaUSA; ^37^ GENOMEDCenter for Medical GeneticsUniversity of AntwerpAntwerpBelgium

**Keywords:** Dravet syndrome, epilepsy, genetic screening, next‐generation sequencing, Sanger sequencing

## Abstract

**Background:**

Sanger sequencing, still the standard technique for genetic testing in most diagnostic laboratories and until recently widely used in research, is gradually being complemented by next‐generation sequencing (NGS). No single mutation detection technique is however perfect in identifying all mutations. Therefore, we wondered to what extent inconsistencies between Sanger sequencing and NGS affect the molecular diagnosis of patients. Since mutations in *SCN1A*, the major gene implicated in epilepsy, are found in the majority of Dravet syndrome (DS) patients, we focused on missed *SCN1A* mutations.

**Methods:**

We sent out a survey to 16 genetic centers performing *SCN1A* testing.

**Results:**

We collected data on 28 mutations initially missed using Sanger sequencing. All patients were falsely reported as *SCN1A* mutation‐negative, both due to technical limitations and human errors.

**Conclusion:**

We illustrate the pitfalls of Sanger sequencing and most importantly provide evidence that *SCN1A* mutations are an even more frequent cause of DS than already anticipated.

## Introduction

When it comes to genetic screenings, Sanger sequencing has long been considered the gold standard and is still widely performed. However, next‐generation sequencing (NGS) is becoming steadily implemented nowadays, both in research and in clinical diagnostic settings. Whereas Sanger sequencing targets only one gene at a time, making it a very time and cost‐consuming method, NGS technologies can analyze a set of genes, an exome, or even a genome in a single sequencing run. This enormous advantage has led to the widespread implementation of different NGS platforms in genetic centers (Sisodiya 2015). It is well‐known that no single mutation detection technique is perfect in identifying all the mutations. Therefore, we wondered to what extent negative findings on Sanger sequencing turn out to be false negative when subsequently analyzed by NGS. To answer this question, we focused our study on screenings of the *SCN1A* gene (OMIM: #152389) in Dravet syndrome (DS), one of the genetically most homogeneous epilepsy syndromes.

DS is among the best defined and most extensively studied entities within the epileptic encephalopathies. Clinically, the disease is characterized by a seizure onset in the first year of life, usually around six months. Seizures at onset are fever sensitive, and mostly consist of generalized or unilateral, often prolonged, clonic, and tonic–clonic seizures. As the disease progresses, afebrile seizures co‐occur, and other seizure types such as myoclonic seizures, atypical absences, and focal seizures become more prominent (Dravet 2011). Seizures usually are resistant to currently available antiepileptic drugs. The development of patients with DS is initially normal. During the second year of life however, developmental delay and other neurological defects become apparent (Brunklaus et al. 2012).

The most important gene implicated in DS is *SCN1A,* encoding the alpha subunit of the neuronal voltage‐gated sodium channel Na_v_1.1. About 70% to 80% of DS patients are shown to carry an *SCN1A* mutation of which 90% occur de novo (Claes et al. 2001; Depienne et al. 2009a). Single nucleotide substitutions, small indels, and even whole gene deletions have been reported with at least 1257 different mutations described to date (Suls et al. 2006; Zuberi et al. 2011; Meng et al. 2015). These mutations occur randomly throughout the gene, without the presence of mutational hotspots. Recently, mutations in several other genes including *PCDH19, GABRG2*,* CHD2*, and *HCN1* have been associated with a DS phenotype. However, each of these genes only has a small contribution.

*SCN1A* mutations can also be found in a few other epilepsy syndromes that show some clinical similarities to DS, such as myoclonic atonic epilepsy (MAE) and genetic epilepsy with febrile seizures plus (GEFS+). The mutation yield in these syndromes is however much lower, ranging from a few percent up to 10% (Hirose et al. 2013).

Despite the significant contribution of genetic alterations in *SCN1A* to DS, a subset of patients remain without a genetic diagnosis after testing of *SCN1A* with Sanger sequencing. These patients may harbor mutations in one of the “minor” Dravet genes but could also represent *SCN1A* false‐negative cases that are carrying an *SCN1A* mutation missed by Sanger sequencing. Within our EuroEPINOMICS‐RES consortium, we performed whole‐exome sequencing (WES) on 31 DS trios (patient and healthy parents; cohort previously described (Syrbe et al. 2015)) identifying *SCN1A* mutations in eight patients considered *SCN1A* mutation‐negative upon Sanger screening (unpublished data). This observation shows the limitations of Sanger sequencing, but most importantly indicates that *SCN1A* mutations are an even more frequent cause of DS than is generally accepted.

After our prospective EuroEPINOMICS‐RES consortium study we conducted an additional retrospective study to collect additional information on missed *SCN1A* mutations and explored why all these mutations were originally missed.

## Materials and Methods

The study was approved by the local ethics committees of participating centers. The protocol and procedures employed were reviewed and approved by the appropriate institutional review committee. Informed consent was obtained for the patients described in this study. The followed procedures were in accordance with the ethical standards of the responsible committees on human experimentation and with the Helsinki Declaration of 1975, as revised in 2008.

We sent out a survey to 16 genetic centers performing *SCN1A* screening on a diagnostic and/or research basis, to collect information on *SCN1A* mutations (RefSeq NM_001165963.1) that were searched for by Sanger sequencing, but were only subsequently identified by NGS. This study was broader than DS and included all phenotypes related to *SCN1A*. In order to compare the results of these genetic tests, we decided to only analyze detection errors of point mutations or small insertions or deletions. Partial/whole gene deletions and occasionally duplications of *SCN1A* are also a well‐documented cause of DS. These structural alterations can be missed by Sanger sequencing and may undoubtedly contribute to the group of *SCN1A* false‐negative cases. This was confirmed in the EuroEPINOMICS‐RES cohort where testing with array‐CGH detected two deletions in the remaining 23 *SCN1A*‐negative patients (unpublished data). In the retrospective study, we did not include copy number variants since calling these variants from NGS data is still challenging and clear comparisons between the results of the techniques can thus not be made. With the questionnaire, we specifically asked for information on the sequencing techniques, the reasons for missing the mutation, the setting (diagnostic vs. research), and the date of the screening. All mutations have been submitted to the *SCN1A* database (http://www.gzneurosci.com/scn1adatabase/index.php).

## Results

We received a response from 16 different genetic centers, of which 13 had one or more patients to include. In the retrospective study, we collected information on 20 additional patients harboring an *SCN1A* mutation that was missed by Sanger sequencing but confirmed by NGS and one patient in whom an *SCN1A* mutation was detected by Sanger sequencing that was missed in a WES study looking for modifiers (Table 1). Eighteen patients were diagnosed with DS, two other patients presented a phenotypically related epilepsy syndrome (GEFS+, MAE) and one patient had an epileptic encephalopathy without further phenotypical information (Table 1). Seven of the patients have previously been reported (patient 2, 16, 17, 18, 19, 20, 29) (Table 1).

**Table 1:** Genetic and clinical information of the *SCN1A* mutations reported in this study as well as the setting and the date the samples were screened

Patient	Mutation		Inheritance	Novel^f^	Phenotype	Setting negative screening	Setting positive screening	Date negative screening	Date positive screening
	cDNA	Protein							
1^a^	c.1121C>A	p.Ser374Tyr	*De novo*	Yes	DS	Research	Research	2012	2012
2^a^	c.664C>T	p.Arg222*	*De novo*	No	DS	Research	Research	2006	2012
3	c.4002+1G>A		*De novo*	No	DS	Diagnostic	Diagnostic	2013	2014
4	c.4284+1G>A		*De novo*	No	DS	Diagnostic	Research	2007	2013
5	c.1178G>A	p.Arg393His	*De novo*	No	DS	Diagnostic	Research	2009	2011
6	c.5269G>A	p.Gly1757Arg	*De novo*	Yes	DS	Diagnostic	Research	2010	2013
7	c.5656C>T	p.Arg1886*	*De novo*	No	DS	Diagnostic	Research	2010	2013
8	c.53_55delCCA	p.Thr18del	Unknown	Yes	DS	Diagnostic	Diagnostic	2010	2015
9	c.602+1G>C		Unknown	Yes	DS	Diagnostic	Diagnostic	2010	2015
10^a^	c.5461C>T	p.Gln1821*	*De novo*	No	DS	Research	Research	2011	2013
11	c.379C>T	p.His127Tyr	*De novo*	Yes	GEFS+	Diagnostic	Research	2007	2013
12	c.302G>A	p.Arg101Gln	*De novo*	No	DS	Diagnostic	Diagnostic	2013	2015
13^a^	c.4853‐1G>A		*De novo*	Yes	DS	Research	Research	2011	2013
14^a^	c.3439G>T	p.Glu1147*	*De novo*	No	DS	NA	Research	NA	2012
15	c.302G>A	p.Arg101Gln	*De novo*	No	DS	Diagnostic	Research	2007	2013
16^c^	c.5195C>T	p.Pro1732Leu	*De novo*	No	DS	Diagnostic	Research	2010	2013
17^d^	c.2044‐1G>A		*De novo*	No	DS	Research	Research	2002	2011
18^c^^,^ a	c.2590‐8T>G		*De novo*	No	DS	Diagnostic	Research	2010	2013
19^b^	c.1178G>A	p.Arg393His	*De novo*	No	DS	Diagnostic	Research	2009	2011
20^e^^,^ a	c.3452C>G	p.Ser1151*	*De novo*	No	DS	Research	Research	2010	2012
21	c.4889T>G	p.Val1630Gly	*De novo*	Yes	DS	Diagnostic	Research	2011	2013
22	c.2589+3A>T		*De novo*	No	DS	Research	Research	2013	2005
23^a^	c.4786C>T	p.Arg1596Cys	*De novo*	No	DS	Diagnostic	Research	2008	2013
24^a^	c.5347G>A	p.Ala1783Thr	*De novo*	No	DS	Research	Research	2010	2012
25	c.5536_5539delAAAC	p.Lys1846Serfs*11	Unknown	No	Unspecified EE	Diagnostic	Research	2008	2012
26	c.5771delG	p.Arg1924Leufs*8	*De novo*	Yes	DS	Diagnostic	Diagnostic	2014	2014
27	c.4573C>T	p.Arg1525*	*De novo*	No	MAE	Diagnostic	Diagnostic	2006	2013
28	c.1129C>T	p.Arg377*	Absent in mother, father not tested	No	DS	Research	Research	2004	2013
29^d^	c.383C>A	p.Ser128*	*De novo*	No	DS	Diagnostic	Diagnostic	2011	2011

DS: Dravet syndrome; EE: epileptic encephalopathy; GEFS+: genetic epilepsy with febrile seizures plus; MAE: myoclonic atonic epilepsy; NA: not applicable; WES: whole‐exome sequencing. Accession number for *SCN1A*: RefSeq NM_001165963.1, NP_001159435.1. Seven patients have previously been reported.

a These eight patients are part of the EuroEPINOMICS‐RES cohort.

b Lemke et al., 2012.

c Bayat et al., 2015.

d Carvill et al., 2014.

e Gaily et al., 2013.

f Based on the *SCN1A* database (http://www.gzneurosci.com/scn1adatabase/index.php) and the published papers mentioned in this manuscript.

When comparing the technologies, 28 mutations were detected using NGS after prior screening with Sanger sequencing was reported to be negative. One mutation was initially found by Sanger sequencing but subsequent WES (aiming to investigate additional genetic modifiers) failed to identify the mutation. Reasons for missing these mutations can be classified into three categories (Table 2): mutations were missed due to (1) human errors, (2) technical problems of the screening technique, and (3) unknown reasons.

**Table 2:** Overview of the different reasons that *SCN1A* mutations were missed in a genetic screening

Patient	Negative screening	Positive screening	Reason that the mutation was missed
			**Human error**
1	Sanger	NGS: WES	Missed by the person performing the mutation analysis (visual inspection of Sanger traces)
2^a^	Sanger	NGS: Gene panel	Missed by the person performing the mutation analysis (visual inspection of Sanger traces)
3	Sanger	NGS: Gene panel	Missed by the person performing the mutation analysis (visual inspection of Sanger traces)
4	Sanger	NGS: WES	Missed by the person performing the mutation analysis (visual inspection of Sanger traces)
5	Sanger	NGS: WES	Missed by the person performing the mutation analysis (visual inspection of Sanger traces)
6	Sanger	NGS: Gene panel	Missed by the person performing the mutation analysis (visual inspection of Sanger traces)
7	Sanger	NGS: Gene panel	Missed by the person performing the mutation analysis (visual inspection of Sanger traces)
8	Sanger	NGS: Gene panel	Missed by the person performing the mutation analysis (visual inspection of Sanger traces)
9	Sanger	NGS: Gene panel	Missed by the person performing the mutation analysis (visual inspection of Sanger traces)
10	Sanger	NGS: WES	Error in the primer design: The mutation was located in the primer binding site
11	Sanger	NGS: WES	Error in the primer design: A polymorphism in the primer led to mono‐allelic amplification
12	Sanger	NGS: Gene panel	Error in the primer design: A polymorphism in the primer led to mono‐allelic amplification
13	Sanger	NGS: WES	Error in the primer design: The primer did not cover the whole amplicon (only one direction was sequenced)
14	Sanger	NGS: WES	The patient turned out not to be sequenced
15	Sanger	NGS: WES	Possible sample swap outside the lab
16^b^	Sanger	NGS: Gene panel	Wrong sequencing data assigned to the patient
17^c^	Sanger	NGS: WES	The traces were of bad quality so the Sanger should have been redone
18^b^	Sanger	NGS: Gene panel	Not reported in the diagnostic report as the mutation is located eight base pairs in the intron
19^a^	Sanger	NGS: Gene panel	An adjacent intronic polymorphic deletion led to misalignment of the alleles and uninterpretable data
			**Technical error**
20^d^	Sanger	NGS: WES	The annealing temperature of the primers was too high for the polymerase
21	Sanger	NGS: WES	The mutated peak was too low
22	NGS: WES	Sanger	The mutation is located in a homopolymer stretch
			**Unknown reason**
23	Sanger	NGS: WES	Unable to retrieve the Sanger traces
24	Sanger	NGS: WES	Unable to retrieve the Sanger traces
25	Sanger	NGS: Gene panel	Unable to retrieve the Sanger traces
26	Sanger	NGS: Gene panel	Unable to retrieve the Sanger traces
27	Sanger	NGS: WES	Unable to retrieve the Sanger traces
28	Sanger	NGS: Gene panel	Unable to retrieve the Sanger traces

NGS: next‐generation sequencing; WES: whole‐exome sequencing. Seven patients have previously been reported.

a Lemke et al., 2012.

b Bayat et al., 2015.

c Carvill et al., 2014.

d Gaily et al., 2013.

The most frequent reason for reporting false‐negative results were human errors (19/29; 66%). In retrospect, for nine patients, the mutation was present in the Sanger traces, but was simply overlooked by the person performing the initial analysis. Problems with primer design led to false‐negative results in four patients. The medical report of one patient erroneously stated that he was sequenced although he never was; a sample switch occurred for one patient; one patient was assigned the wrong sequencing data; for one the Sanger sequencing results were of bad quality so the sequencing should have been repeated; and for another patient, the mutation was positioned eight base pairs into the intron and was therefore considered not significant and thus not mentioned in the diagnostic report. Finally, one patient had an intronic deletion leading to misalignment of the reads and consequently uninterpretable data.

In three patients (10%), technical problems led to missing the mutations. One mutation was not identified in the Sanger traces due to the use of an excessively high primer annealing temperature, but was detected by WES and confirmed in a second Sanger sequencing run at a lower temperature. For one patient, the peak of the mutation in the electropherogram was too low to be called as a variant by the analysis software. A first WES run on this sample suggested mosaicism (49 reference reads vs. 18 variant reads), and this was confirmed by a second WES run (169 reference reads vs. 81 variant reads). The mutation detected by Sanger but subsequently missed by WES was an A>T substitution lying in a stretch of adenine nucleotides, creating a long homopolymer knowing to cause problems in variant calling (both false‐positive and false‐negative calls) using NGS sequencing.

For the remaining seven patients, we could not trace the original sequencing reports and were thus unable to identify the exact reason for mutation detection failure.

## Discussion

When comparing sequencing techniques, our data show that Sanger sequencing resulted in 28 false‐negative results while NGS missed one mutation. First of all, it should be noted that these numbers probably give an incorrect impression of the reliability of the different techniques, since our retrospective analysis creates an ascertainment bias toward patients initially screened by Sanger sequencing. Although NGS is the logical next step when Sanger sequencing is negative, few patients will undergo Sanger sequencing after a negative NGS screening, unless there is evidence of low coverage of a particular gene or a particularly convincing phenotype.

Both techniques clearly have their own technical limitations, as illustrated in this study. NGS is known to be superior to Sanger sequencing for the detection of low levels of mutant allele, as seen in mosaicism. A probable mosaic mutation was indeed first missed by Sanger sequencing, but subsequently detected by WES in one patient in this study (patient 21). The importance of germline and somatic mosaicism is well established in a broad range of diseases, including DS (Vadlamudi et al. 2010), and highlights the usefulness of high coverage NGS techniques for mosaic mutation detection. A major weakness of NGS on the other hand is the sequencing of stretches of the same nucleotide, which can lead to homopolymer‐associated insertion and deletion errors due to the nonlinear light response generated by the nucleotide stretches (patient 22). Another disadvantage of NGS is the use of relatively short reads, although read lengths are increasing steadily with advancing NGS techniques. Short reads can lead to problems with mapping quality, especially in repeat regions, which in turn can result in misalignments and misinterpretation of the data (Stranneheim and Lundeberg 2012).

Our study further showed that the majority (19/29) of mutations were missed due to human errors, which in most cases could have been prevented by applying rigorous quality controls. Sample handling and allocation remain error‐prone steps independent of the sequencing technology. In this context, the use of a well‐functioning laboratory information management system (LIMS) is crucial. Keeping track of all the processes and logging every detail may seem very labor intensive, but might eventually save the costs of a potential redundant NGS experiment. In recent years, strict quality control procedures and criteria, including the use of LIMS, have been developed for diagnostic genetic laboratories, and are expected to result in a reduction of human errors. Also the analysis/interpretation process is prone to errors that are more difficult to eradicate. Errors resulting from visual inspection of Sanger traces can be circumvented by using automated variant calling. Errors related to primer design can be overcome by a more careful control of parameters used in software for primer design. Recent years have also brought us more sophisticated *in silico* variant annotation and prediction tools that are greatly aiding in our interpretation of variants, as illustrated for the splice variant in patient 18. Our data show that most mutations were missed during the early implementation of *SCN1A* mutation testing in clinical practice. Nevertheless, even during the last two years false‐negative results were generated in a highly regulated diagnostic setting, which shows that there is still room for improvement of quality control (Table 1).

Identifying *SCN1A* mutations in patients supposed to be *SCN1A*‐negative is not a unique observation of our study but has been described previously. Carvill et al. reported three mutations in 13 patients with DS in whom a previous *SCN1A* screening turned out negative (Carvill et al. 2014). Lemke et al. described two mutations in a cohort of 33 patients with diverse epilepsy phenotypes (Lemke et al. 2012), Bayat et al. mentioned two patients with DS who initially tested negative upon *SCN1A* screening (Bayat et al. 2015) and Gaily et al. reported one such patient (Gaily et al. 2013). Finding a mutation in prescreened and so‐called mutation‐negative patients is also not limited to *SCN1A*, nor the epilepsy field. For example, Klein and colleagues described five kindreds with inherited polyneuropathy in whom WES identified known pathogenic mutations that were initially overlooked by Sanger sequencing, showing this phenomenon to be a general concern for genetic diagnostics (Klein et al. 2014).

In total, we collected 29 *SCN1A* mutations in DS patients erroneously reported as mutation‐negative. This illustrates that the frequency of *SCN1A* mutations in DS is still underestimated and higher than the reported 80%. The identification of an *SCN1A* mutation in 32% (10/31) of DS patients from our “*SCN1A*‐negative” EuroEPINOMICS‐RES consortium study clearly shows that DS is even more genetically homogenous than previously anticipated. That 13 of the 16 participating centers contributed false‐negative cases indicates that missing *SCN1A* mutations occurs regularly. However, the exact frequency could not be determined as in the prospective EuroEPINOMICS‐RES study. Given the variability in data storage procedures of the different genetic centers involved in this study, we were unable to retrieve information on the total number of *SCN1A*‐negative patients that underwent a genetic screening with a second technology.

Given the high genetic homogeneity of DS, first‐line testing for DS should be the search for an *SCN1A* mutation. Whether this is performed using Sanger sequencing or NGS (e.g., a gene panel with a high coverage of *SCN1A*) seems to be of lesser importance as no technique is perfect in identifying all the mutations. In case of a negative *SCN1A* test in a patient with a convincing clinical suspicion of DS, we recommend clinicians to discuss the need to use a second genetic technique and analyze *SCN1A* in depth to be absolutely sure that no mutation is present. It should however be noted that despite the clear genotype–phenotype correlation between *SCN1A* mutations and DS, mutations in several other genes have also been associated with a DS phenotype (Depienne et al. 2009b; Carvill et al. 2014; Nava et al. 2014). Additionally, aside from these “missed” coding mutations, we can expect that mutations in noncoding regulatory regions of *SCN1A* and possibly also epigenetic factors affecting the gene might play a role in the pathogenesis of DS. A negative *SCN1A* screening should therefore not be considered as an exclusion factor for DS.

Finding a mutation and thus providing a clear etiological diagnosis has major implications for the patient and his/her family comprising not only issues related to prognosis and family planning but also interventions toward a more tailored treatment.

## Conflict of Interest

None declared.
